# Ray Optics Model for Optical Trapping of Biconcave Red Blood Cells

**DOI:** 10.3390/mi14010083

**Published:** 2022-12-29

**Authors:** Riccardo Tognato, Philip H. Jones

**Affiliations:** Department of Physics and Astronomy, University College London, Gower St., London WC1E 6BT, UK

**Keywords:** optical tweezers, red blood cell, ray optics

## Abstract

Red blood cells (RBCs) or erythrocytes are essential for oxygenating the peripherical tissue in the human body. Impairment of their physical properties may lead to severe diseases. Optical tweezers have in experiments been shown to be a powerful tool for assessing the biochemical and biophysical properties of RBCs. Despite this success there has been little theoretical work investigating of the stability of erythrocytes in optical tweezers. In this paper we report a numerical study of the trapping of RBCs in the healthy, native biconcave disk conformation in optical tweezers using the ray optics approximation. We study trapping using both single- and dual-beam optical tweezers and show that the complex biconcave shape of the RBC is a significant factor in determining the optical forces and torques on the cell, and ultimately the equilibrium configuration of the RBC within the trap. We also numerically demonstrate how the addition of a third or even fourth trapping laser beam can be used to control the cell orientation in the optical trap. The present investigation sheds light on the trapping mechanism of healthy erythrocytes and can be exploited by experimentalist to envisage new experiments.

## 1. Introduction

Red blood cells (RBCs), or erythrocytes, are the most abundant cell present in the blood of most vertebrates, whose principal function is the delivery of oxygen to peripheral tissues and organs. In humans, mature and healthy RBCs are biconcave disks with a typical diameter of 6–8 μm, and a thickness of 2–3 μm in the periphery, decreasing to 0.8–2 μm in the central dimple [1]. The disk shape minimizes the membrane bending energy, while the excess surface area and membrane elasticity render the cell highly flexible, thereby permitting it to pass through the microvasculature [2]. Variation of the biomechanical properties of RBCs has the potential to cause dysfunctions in the microcirculation, and in the most severe cases capillaries can be entirely obstructed causing tissues necrosis or organ damage and failure. Alteration of the deformability of RBCs can be genetic, such as occurs in the inherited disorders sickle cell disease, hereditary spherocytosis, and elliptocytosis [3], or as a consequence of infection by a pathogen such as malaria [4]. Changes in RBC elasticity have also been correlated to metabolic disorders such as diabetes [5], to radiation treatment [6], and very recently to SARS-CoV-2 infection [7].

Optical tweezers (OTs) are a powerful tool able to confine and manipulate microscopic (and sub-microscopic) dielectric objects using a tightly focused laser beam [8]. The trapping and handling of dielectric particle relies on the forces generated by radiation pressure: in OT, the prevailing force component is the gradient force which draws the particle towards the high intensity region of the focused laser beam. Simultaneously, a scattering force component pushes the particle in the direction of propagation of the light beam. The point in space where the force components cancel out is the point of equilibrium of the trapped particle within the optical trap [9]. Additionally, for non-spherical particles, the shape may give rise to an optical torque, which causes particle rotation and orientational alignment [9].

To date, OTs have been used to trap and manipulate a diverse range of inanimate micro- and nano-particles [10,11,12], in addition to biological samples such as viruses or bacteria [13], and eukaryotic cells [14]. Significantly, during the last two decades, OTs have been demonstrated to be an effective technique to manipulate, investigate, sense, and screen the biomechanical properties of both healthy and unhealthy RBCs [15]. Two main techniques have been employed so far in RBC optical trapping studies. The first method makes use of handles, such as polystyrene or silica microsphere, which are allowed to adhere to the cell membrane and are then held in the OT and so used to trap, manipulate, or stretch the cell [16]. In the second approach, the cell is trapped and manipulated by single or multiple focused laser beams which illuminate the cell directly and without optical damage [15]. In many situations this scheme is advantageous due to its experimental simplicity.

In a typical direct trapping experiment, RBCs are found resting on the surface of a microscope slide or cover slip, with their plane initially transverse to the propagating light beam, from now on referred to as a ‘flat’ configuration. The optical force pulls the RBC into the beam(s) while an optical torque rotates the RBC, such that its plane becomes oriented parallel to the optical axis. This is described as the RBC getting ‘folded’ into an optical trap [15,17], and so, we will refer to this as ‘folded’ configuration for both single- and dual-beam optical tweezers [5,18]. We must point out here that this does not refer to folding of the cell itself, but simply to the orientation with respect to the optical axis. Other applications combine multi-beam optical tweezers with techniques such as Raman spectroscopy. Rusciano et al. reported an example of the application of Raman-Tweezers to the study of RBCs where a four-beam optical tweezers was used to trap the cell while maintaining the flat configuration. Simultaneously, a fifth laser beam with a different wavelength was used to scan the cell to excite the Raman mode of the biomolecule of interest [19].

Although numerous experiments have demonstrated how it is possible to optically trap healthy RBCs, and control their equilibrium configuration by using a different number of laser beams [15], relatively little theoretical work has been performed to explore the trapping mechanism. Grover et al. explored the equilibrium configuration of an RBC trapped in a counter-propagating beam trap [20], however, the trapping mechanism in this case is essentially different from that of OT since it relies on a balance of optical scattering forces for stability. A numerical investigation of the mechanism of trapping is needed to comprehend better how the intricate shape of the RBC influences the trapping in optical tweezers. Moreover, the ability to calculate the optical forces and torques along with the trapping stiffnesses is essential for the experimentalists to optimize experimental parameters before performing experiments on, e.g., RBC deformability.

In this paper we report a numerical investigation of the optical forces and torques acting on a healthy RBC in its native biconcave morphology when directly trapped by both single- and multiple-beam optical tweezers. 

Given the size of the objects, ray optics approximation is the favorite computational methodology to calculate optical forces and torques on the RBC for the easy of the calculations [21]. The ray optics method also permits us to calculate, e.g., stress distribution over the cell surface even for the cells that are not osmotically swollen into a spherical shape [22] that is not possible with a dipole approximation [23]. For this reason, we use ray optics approximation to find the equilibrium position and orientation and to explore the possibilities to control the orientation of the cell. We find that the characteristic biconcave shape of the healthy RBC plays a significant role in determining the stability of the trap, and that the effective weight of the cell cannot be neglected when finding the equilibrium location. We show how the two-beam trap commonly used in RBC experiments provides rotational stability about the optical axis, and demonstrate how the introduction of additional laser beams provides a route to controlling cell orientation.

## 2. Model

In our model, a three-dimensional Cartesian (*x*, *y*, *z*) co-ordinate system is considered. The light beams possess a Gaussian intensity distribution and propagate along +*z*. The wavelength of the trapping laser beam is chosen to be λ = 1.064 μm, and the numerical aperture (NA) of the objective lens chosen to be 1.3, as these values are comparable with a large number of reported RBC trapping experiments [15,23]. The power of the beam is chosen to be 5 mW, since higher powers may deform the cell, and prolonged exposure to higher powers may damage it [22]. In the multi-beam optical trap calculations, a power of 5 mW is assigned to each beam.

To be comparable to experiments, the RBC is considered to be immersed in a water-based medium with refractive index *n*_1_ = 1.33. The cell membrane is modelled as a single net line, and the interior of the RBC is assumed to be filled by a non-absorbing medium with isotropic physical properties. The refractive index of the cell interior is *n*_2_ = 1.38, and the cell’s mass density is 1.11 g/cm^3^ [24]. The RBC is considered to be in its healthy biconcave disk conformation described by the parametric equation introduced by Evans and Fung [1]:

(1) $${\left(\frac{z_{cell}}{r}\right)}^{2}-\left(1-\frac{\left(x_{cell}{}^{2}-y_{cell}{}^{2}\right)}{r^{2}}\right){\left(C_{0}{\;+\;C}_{1}\left(\frac{x_{cell}{}^{2}+y_{cell}{}^{2}}{r^{2}}\right){+C}_{2}\left(\frac{x_{cell}{}^{2}+y_{cell}{}^{2}}{r^{2}}\right)\right)}^{2}=0,$$

where *C*_0_, *C*_1_ and *C*_2_ are the shape coefficients for the RBC. *C*_0_, *C*_1_ and *C*_2_ can be tuned to obtain the specific morphology that describe a particular experimental condition. We use the relations introduced by Valchev et al. given in Equation (23) in the respective reference [25]. In our model the cell radius, *r* = 3.91 μm, the minimum thickness of the cell is 0.81 μm, while the thickest portion, located at 2.76 μm from the cell’s center, has a thickness of 2.52 μm. For the low optical powers used in the calculations the RBC is always considered to be non-deforming in response to radiation pressure. 

The characteristic size of the trapped object is such that the ray optics (RO) criterion is satisfied [26], thus, the optical beam impinging on the RBC can be decomposed in a bundle of light rays provided with appropriate intensity and direction. Each single light ray is provided with a momentum *p* = (*nP*)/*c*, where *P* is the power of the beam, *n* is the refractive index of the medium in which the ray is travelling, and *c* is the speed of light. In RO, the optical force exerted by a light ray on the object’s center of mass is calculated as the rate of change of momentum between the incoming and the outgoing rays at a refraction or reflection event [26]:

(2) $$F_{\mathrm{ray}}=\frac{n_{1}P_{i}}{c}{\hat{r}}_{i}-\frac{n_{1}P_{r}}{c}{\hat{r}}_{r^{-}}\overset{+\infty }{\underset{n=1}{\sum }}\frac{n_{i}P_{t,n}}{c}{\hat{r}}_{i,n,}$$

where *P_i_*, *P_r_* and *P_t,n_* are the powers of the incident, reflected and every successive reflected/transmitted ray. The partition of the incident power between reflected and transmitted rays is calculated according to Fresnel’s coefficients [26]. The total optical force acting on the center of mass of the trapped object is then calculated as the sum of the contribution due to each single light ray [26]:

(3) $$F_{\mathrm{GO}}=\underset{m}{\sum }F_{\mathrm{ray}}^{\left(m\right)}=\underset{m}{\sum }[\frac{n_{1}P_{i}^{\left(m\right)}}{c}{\hat{r}}_{i}^{\left(m\right)}-\frac{n_{1}P_{r}^{\left(m\right)}}{c}{\hat{r}}_{r,0}^{\left(m\right)}-\overset{+\infty }{\underset{n=1}{\sum }}\frac{n_{i}P_{t,n}^{\left(m\right)}}{c}{\hat{r}}_{t,n}^{\left(m\right)}].$$

However, since in this case the light rays are interacting with a non-spherical particle, significant torques may also arise. The torque generated by a single light ray is calculated as the difference of the angular momentum associated with the incoming ray and that of the outgoing rays [26]:

(4) $$T_{\mathrm{ray}}^{\left(m\right)}=\left(P_{0}-C\right)\times \frac{n_{i}P_{i}^{\left(m\right)}}{c}{\hat{r}}_{i}^{\left(m\right)}-\left(P_{0}-C\right)\times \frac{n_{i}P_{r}^{\left(m\right)}}{c}{\hat{r}}_{r,0}^{\left(m\right)}-\overset{+\infty }{\underset{n=1}{\sum }}\left(P_{n}-C\right)\times \frac{n_{i}P_{t,n}^{\left(m\right)}}{c}{\hat{r}}_{t,m}^{\left(m\right)}$$

where **C** is the center of mass of the object, **P**_0_ is the incidence point of the incoming ray, and **P**_n_ are the scattering points of the subsequently scattered rays. Therefore, the total torque acting on the object is the sum of the contribution of each single light ray [26].

For biological samples such as RBC, the fraction of power that is reflected after a scattering event is very low, and to a good approximation only the first two scattering events may be considered [27]. This is achieved by truncating the sum on the right-hand side of Equations (2) and (4) at the second term. 

Given the generality of the theoretical framework introduced in [26], our model is well fitted within this context. A freely available software namely Optical Tweezers in Geometrical (OTGO) implements (in MatLab, version R2019a) the theory reported in [25] in a modular object oriented software [28]. In the available distribution of OTGO only simple geometrical shapes, such as spheres or ellipsoids, are present, but the modularity of the software makes it easy for researchers to implement new objects describing more complex shapes. The initial implementation of new objects requires some advanced programming skills, but the later usage is straightforward and facilitated by the presence of a series of examples. For these reasons, we decided to incorporate our model for biconcave disk RBC in a new object in OTGO and perform the entire set of numerical experiments with this software. 

## 3. Results and Discussion

### 3.1. Single-Beam Optical Tweezers

**Figure 1:**
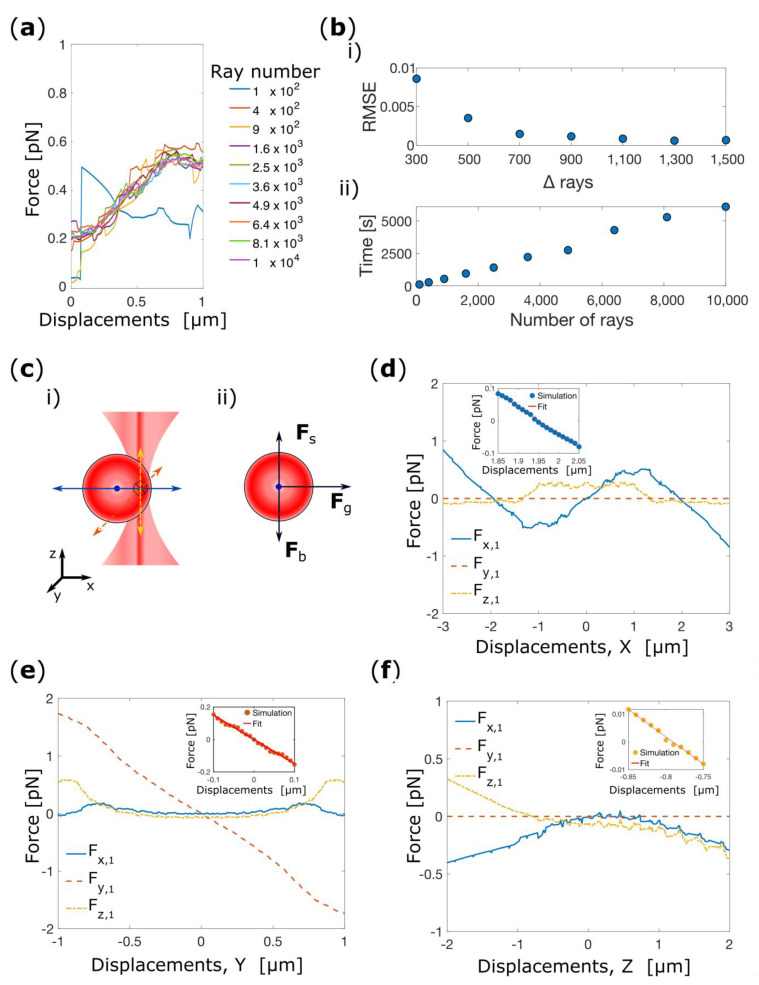
(**a**) Simulation of $F_{tot}\left(x\right)$ as a function of the number of light ray used in the simulation. (**b**) Residual mean squared error between $F_{tot}\left(x\right)$ calculated with an increasing number of light rays (**i**) and time required to simulate 601 points as a function of the number of light rays (**ii**). (**c**) Schematic depiction of the translation of the RBC (**i**) and respective free body diagram (**ii**) $F_{g}$ gradient force, $F_{s}$ scattering force and $F_{b}$ effective weight. Cartesian components of the total optical force acting on the center of mass of the RBC as a function of the cell’s displacements in respect to the center of the optical trap for displacements along *x* (**d**), *y* (**e**) and *z* (**f**). The laser beam power is kept constant at 5 mW for each simulation, and the cell is moved with steps of 10 nm along each direction. The inserts show the linear fit of the force-displacements curve near the equilibrium point.

Prior to our systematic investigation of the RBC trapping behavior, we determine the convergence of the simulation as a function of the number of light rays used to describe the light beam. To do so, we simulate numerically the total optical force ($F_{\mathrm{tot}}$) acting on the centre of the cell as function of the cell displacements from the origin of the reference frame along the *x*-direction ($F_{\mathrm{tot}}\left(x\right)$). To evaluate the convergence, $F_{\mathrm{tot}}\left(x\right)$ is simulated starting from 10^2^ light rays up to 10^4^ light rays. Figure 1a shows $F_{\mathrm{tot}}\left(x\right)$ as a function of the number of rays for cell displacement between 0 and 1 μm. Here, it can be seen that $F_{\mathrm{tot}}\left(x\right)$ rapidly converges as the number of light rays increases. At each step we calculate the mean squared deviation between the successive ray traces. This quantity decreases rapidly to <1 ×10^−3^ pN when the number of rays used exceeds 1.6 × 10^3^ as shown in Figure 1b(i). Moreover, since we also find that the computation time increases linearly with the number of light rays used in the simulation, as shown in Figure 1b, for the remainder of the simulations we use 1.6 × 10^3^ rays in each beam as an acceptable compromise between convergence to the large ray number solution and excessive computation time. All calculations were performed on a standard laptop equipped with an Intel Core i5 (dual core, 2.7 GHz) and 8 Gb of RAM.

We first consider an RBC trapped by a single-beam optical tweezers (SBOT) in its folded configuration. This example serves to both illustrate our methodology, and to highlight some important effects on the light rays (and the consequences for the optical force induced) that arise from the complex biconcave disk shape of the erythrocyte. The RBC is firstly displaced along the *x*-direction (i.e., the direction perpendicular to the beam propagation in the plane of the cell) between −3 μm and +3 μm with steps of 0.01 μm as schematically depicted in Figure 1c(i). After each step, the gradient and scattering forces in all three dimensions are calculated to derive the force-displacement curve. In the calculations we also include the effective weight of the cell acting along the negative *z*-direction (Figure 1c(ii)):

(5) $$F_{b}=-V_{\mathrm{cell}}\left(\rho_{RBC}-\rho_{w}\right)\hat{z}.$$

Considering a cell volume of 94 × 10^−18^ m^−3^ [1], we find $F_{b}$ = −0.1 pN. Inclusion of the effective weight will prove to be crucial for the axial trapping stability of the cell.

Figure 1d shows the total optical force in terms of the three Cartesian components as a function of the displacement of the cell’s center of mass (CM) from the origin (the geometrical focal point of the trapping beam). For displacements in the *x*-direction the *y*-component of the forces is zero, whereas significant forces arise along *x* and *z*-direction. *F_z_*_,1_ arises from the scattering force of the trapping laser and acts to push the cell in the *z*-direction. Transverse trapping in the *x*-direction is demonstrated by the form of *F_x_*_,1_. Around the origin the force-displacement curve has positive gradient, indicating that the cell is pushed away from the beam axis, until it finds equilibrium positions where *F_x_*_,1_ vanishes with negative gradient at *x*_eq,1_ = ±1.95 μm. This can be understood as a consequence of the biconcave disc shape of the RBC, since the cell can maximize the overlap of the cell volume with the beam when the beam focus is near the thickest part of the cell. Around the equilibrium position, *x*_eq,1_, the force-displacement curve can be well approximated as an Hookean spring can be well approximated as an Hookean spring (F = −k·∆x), at least small displacement. Following this approximation, fitting a line to the approximately linear part of the graph in the region of the equilibrium positions one gets a (power normalized) spring constant of *k*_x,1_ = 0.17 pN·μm^−1^·mW^−1^.

Subsequently, the RBC is placed at *x*_eq,1_ in the folded configuration. The force-displacement curve is calculated for displacements along the *y*-direction (i.e., normal to the plane of the cell), as shown in Figure 1e (note the smaller range of displacements compared to Figure 1d, since the cell is much thinner in this direction). Here, it can be seen that the optical force in the *y*-direction, *F_y_*_,1_, decreases linearly within the interval examined, and from a linear fit to the data in this interval we extract the trap stiffness in this direction, *k*_y,1_ = 0.30 pN·μm^−1^·mW^−1^ (inset of Figure 1e).

The RBC is then placed at (*x*_eq,1_, *y*_eq,1_) in the folded configuration, and the optical forces are calculated for displacement along *z*, shown in Figure 1f. For this direction it is only when the effective weight of the cell is included that a trapping behavior emerges, that is the total force *F_z_*_,1_ = 0 with a negative slope around an equilibrium position, located at *z*_eq,1_ = −0.8 μm. The power normalized trap stiffness obtained by a linear fit to the force-displacement curve in this region is *k_z_*_,1_ = 0.039 pN·μm^−1^·mW^−1^. 

**Figure 2:**
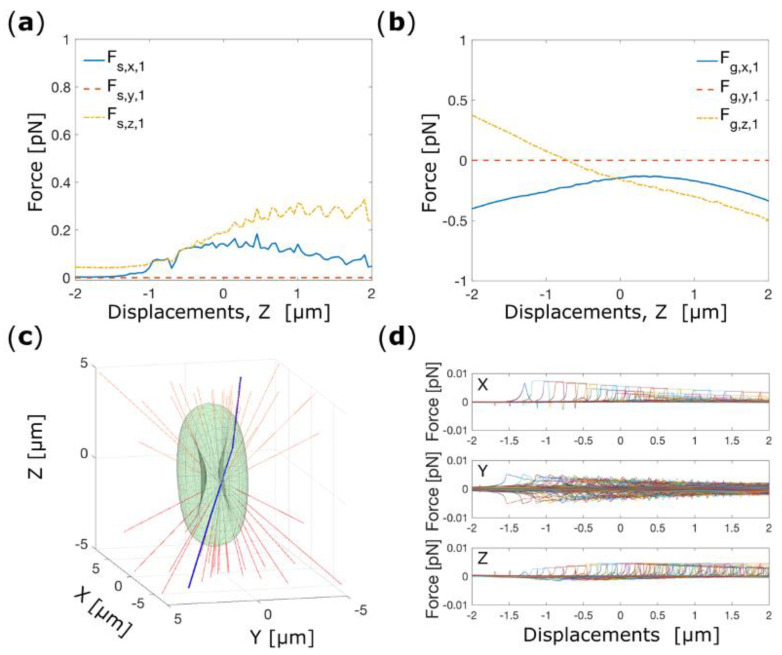
(**a**) Cartesian component of the scattering force as a function of the translation along the *z*-axis. (**b**) Cartesian components of the gradient force acting on the center of mass of the RBC as a function of the cell’s displacements along the *z*-axis. (**c**) Ray traces for a cell placed in the folded position and located at (*x*_eq,1_, *y*_eq,1_, *z*_eq1_). The blue ray indicates the light ray that exerts the highest force on the cell. (**d**) Total optical force exerted by each single light ray on the center of mass of the cell.

The form of the force-displacement curves *F*_x,1_(*z*) and *F_z_*_,1_(*z*) can be understood by decomposing the total force into the scattering (*F*_s_) and gradient (*F*_g_) components, shown in Figure 2a,b. Both *x*- and *z*-components of the scattering force are positive over the range of *z*-displacements investigated. The *x*-component of the gradient force is negative over the range, whereas the *z*-component changes sign. The resultant forces cancel out over a region centered on *z* = 0. This is significant for axial trapping since although the force is zero the gradient of force, and hence the trap stiffness is negligible It is only with the inclusion of the effective weight of the cell that an equilibrium position with non-zero force gradient is found slightly displaced from *z* = 0.

The behavior of the scattering force is a consequence of the shape of the cell, and in particular the ‘dimple’ (concave) region. When the cell is located at the equilibrium position (*x*_eq,1_, *y*_eq,1_, *z*_eq1_) a significant fraction of the rays is incident on the cell in the region of the dimple, and at such an angle that they undergo total internal reflection, shown in Figure 2c, which contributes to a greater scattering force. Due to symmetry in the *x-z* plane the *y*-components cancel out, but in the *x*- and *z*-directions a significant contribution remains preventing stable trapping.

**Figure 3:**
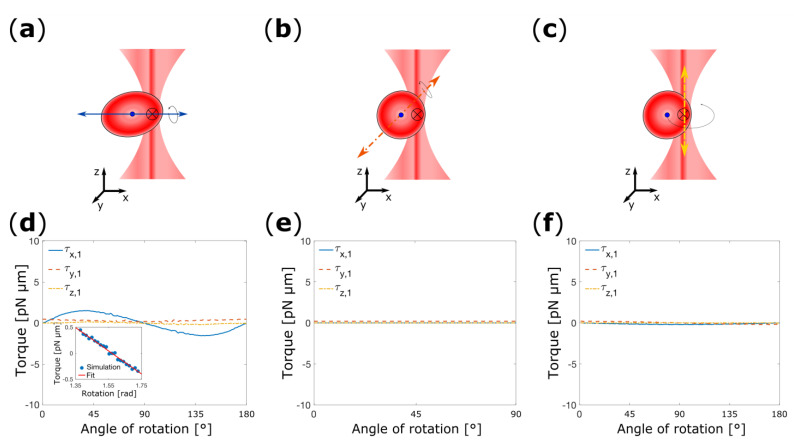
(**a**) Schematic depiction of cell rotation around an axis passing through the center of mass of the RBC and parallel to the *x*-axis. (**b**) Schematic depiction for cell rotation around the *y*-axis. (**c**) Schematic representation of the cell’s rotation around the *z*-axis. The black arrows indicate the direction of rotation. Torque-rotation plot for rotation around *x* (**d**), *y* (**e**), and *z* (**f**). The insert shows the fitting of the optical torques to obtain the rotational tapping stiffness.

Non-spherical objects such as RBCs are subjected also to significant torques which maintain orientation in the trap [29]. To investigate how a single-beam optical tweezers rotationally confines an RBC, we place the cell at (*x*_eq,1_, *y*_eq,1_ and *z* = 0), and we analyze the possible rotation as illustrated in Figure 3a–c. In a single-beam OT, the RBC finds its equilibrium configuration when the cell’s plane is parallel to the optical axis, which implies that a significant restoring torque confines the RBC in its folded configuration. We choose to start the investigation for rotation around the *x*-axis, rotating the cell in the interval 0–180° (where 0° corresponds to the RBC in its flat configuration) with angular steps of 1° and calculating the optical torques (τ) after each step.

Figure 3d shows the torque components acting on the cell while rotating through the interval 0–180°. Here, it can be seen that the cell has an unstable equilibrium with respect to rotations about the *x*-axis when the plane of the cell is perpendicular to the beam (the ‘flat’ configuration). At other angles the cell experiences a torque that rotates it towards the angle 90° (the ‘folded’ configuration). Around this orientation the torque-angle curve is approximately linear, and a fit produces a (power normalized) torque constant *k*_α,1_ = 0.447 pN·μm·rad^−1^·mW^−1^, (inset Figure 3d). The calculations show no other torque components arising from rotations about the *x*-axis exist, and nor do any aligning torques exist for rotations about the *y* or *z* axes. The latter is significant as it implies that the trapped cell is free to rotate around the beam propagation direction.

### 3.2. Multiple-Beam Optical Tweezers

**Figure 4:**
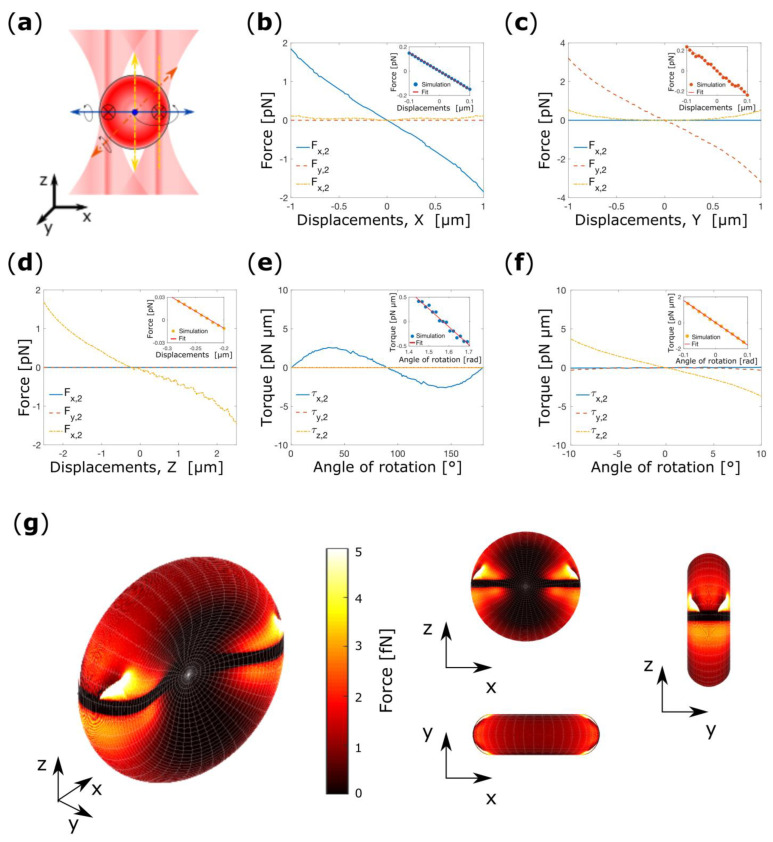
(**a**) Schematic depiction of double-beams optical tweezers. The colored arrows indicate the possible translation of the cell, while the black arrows highlight the possible rotation around the axes represented by the dashed line. (**b**–**f**) Optical forces and torques acting on healthy RBC for a dual-beams optical tweezers. Cartesian components of the total optical force acting on the center of mass of the RBC as a function of the cell’s displacements in respect to the center of the optical trap for displacements along *x* (**b**), *y* (**c**) and *z* (**d**), and Cartesian component of the optical torques for cell rotation around *x* (**e**), and *z* (**f**). The inserts show the linear fit to the approximately linear portion of the force-displacements and torque-rotation curve. In (**g**) are reported the distribution of optical forces generated by the two-beam trap on the surface of the RBC. Note, that the reported forces are solely the normal force at each point.

Now we have thoroughly analyzed the optical trapping of a RBC with a single beam OT, we turned our attention to more complex beam configurations that are also used in experiments. Firstly, we consider an optical trap composed by two beams: double beam optical trap (DBOT). We simulate the forces for the beam configuration used by Agrawal et al. [5] for trapping and stretching RBCs. In this case, the foci of the two beams are positioned 5.06 μm apart along the *x*-axis. As in the case of single beam OT, experiments have shown that in double-beam OT, the RBC finds a trapping equilibrium in its folded configuration [5]. However, by contrast with single-beam OT, in the double-beam OT an additional degree of confinement should arise for rotational motion. The presence of a second beam should confine the cell also for rotations around the *z*-axis, while it is expected that the rotational confinement around *x* and *y* should be unaltered, Figure 4a. 

Figure 4b–f shows the results of the simulations for a DBOT, and in Table 1 are reported the results of the numerical simulation. In a DBOT, the center of mass of the RBC is confined at the origin of the *x*-*y* plane (trap center, *x*_eq,2_ = *y*_eq,2_ = 0 μm) by the synergistic actions of the two beams, Figure 4b,c. Fitting of the linear portion of the graphs produces the (power normalized) spring constants k_x,2_ = 0.15 pN·μm·mW^−1^ and k_y,2_ = 0.24 pN·μm·mW^−1^ (insets Figure 4b,c). In the axial direction the RBC finds its equilibrium for slightly negative z (z_eq,2_ = −0.230 μm), Figure 4d. Fitting a line to the linear portion of *F*_z,2_(*z*) we extract the spring constant k_z,2_ = 0.05 pN·μm·mW^−1^. As may be anticipated, the spring constants in the three directions are not symmetric, and the simultaneous action of the two beams doubles the values of the trap stiffnesses compared to the single-beam case.

Having determined the point of translational equilibrium of a RBC in DBOT, we focus our attention to the analysis of rotational confinements. We proceed by placing the RBC at (x_eq_, y_eq_, z_eq_) and we rotate the cell as schematically depicted in Figure 4a. We start by rotating the cell around the *x*-axis between 0–180° with steps of 1°, where, as before, 0° corresponds to a RBC in its ‘flat’ configuration. After each angular displacement we calculate the optical torques experienced by the cell. Figure 4e shows the Cartesian components of the optical torques as a function of the angle of rotation around the *x*-axis. It can be seen that the cell has a point of unstable equilibrium for 0° (‘flat’ configuration), while for other rotations a significant torque induces the rotation of the cell towards 90° (‘folded’ configuration). In the vicinity of 90°, the torque-rotation curve is approximately linear, and a fit gives a (power normalized) torque constant k_α,2_ = 0.37 pN·μm·rad^−1^·mW^−1^, (inset Figure 4e).

The analysis for rotation around *y*-axis reveals there are no torques acting on the cell as my be expected from symmetry (not shown). Figure 4d shows the cartesian components of the optical torques for a RBC rotated around the *z*-axis between −10°–10°, and where 0° corresponds to a cell oriented parallel to the plane containing the two foci and the trapping axis. In this rotation interval, the RBC experiences a torque that pushes it back towards 0°, with a (power normalized) torque constant k_γ,2_ = 1.73 pN·μm·rad^−1^·mW^−1^, (inset Figure 4f). This is particularly important since suggests that the cell is not free to rotate around the *z*-axis as it is for a single-beam case, and hence the DBOT maintains the orientation of the cell.

Successively, we test the ability of our model in determining the optical forces distribution over the surface of a healthy erythrocytes, Figure 4g. In particular, we calculate the force component normal to the RBC surface that each light ray exerts on the surface ($F_{n}=F_{ray}\cdot \hat{n}$). As expected, the force distribution profile is completely symmetrical in respect to the *x*- and *y*-direction, but it is not symmetrical in respect to the *z*-direction Figure 4g. The force distribution profile shows symmetric peaks on the upper portion of the cell. These peaks correspond, approximately, to the position where the cell is trapped and are due to the combination of rays coming from the outermost part of the beam, and to rays coming from the center of the beam. On the one hand, our method shows good agreement with more complicated computational methodology based on the wave optics theory [30]. On the other hand, our approach shows a substantial advancement in the current methods used for calculating optical forces on healthy biconcave RBC with the ray optics approximation. In fact, our model is not restricted to the fully swollen RBC (i.e., spherical particle) [22], but can be used for RBC in biconcave disk shape, and, by tuning the shape parameters, also on a different range of intermediate shape between the native morphology and the fully swollen one. 

**Figure 5:**
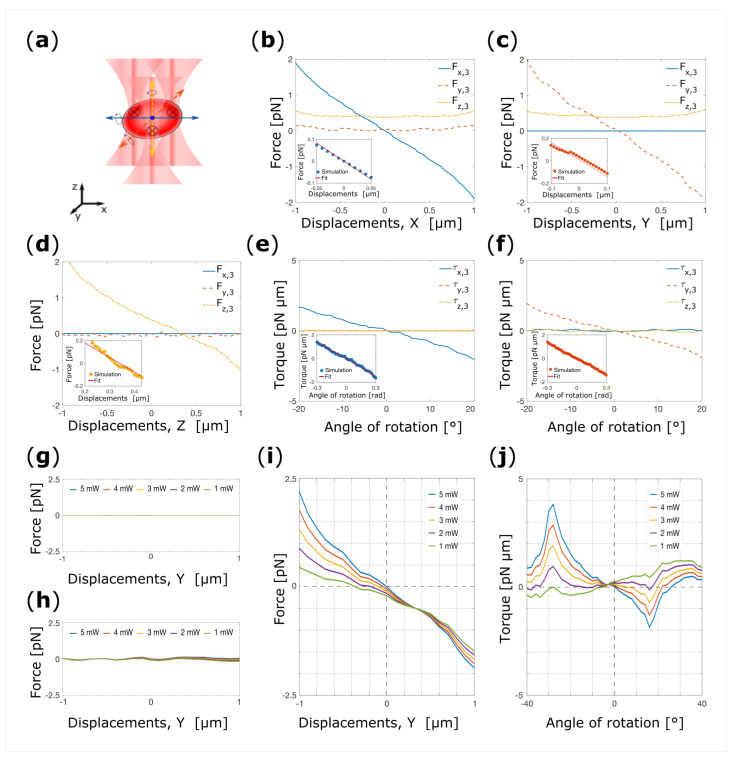
(**a**) Schematic representation of the triple-beam optical tweezers. The arrows indicate the possible translations and rotations of the cell. Cartesian components of the total optical force acting on the center of mass of the RBC as a function of the cell’s displacements from the center of the trap along *x* (**b**), *y* (**c**) and *z* (**d**). Torque-rotation curves for cell rotation around *x* (**e**), and *y* (**f**). The inserts show the linear fit to the approximately linear portion of the force-displacements and torque-rotation curve. Force-displacements curves for cell displacements along the *x*-direction (**g**), *z*-direction (**h**), and *y*-direction (**i**) while in (**j**) is reported the torque-rotation curves. In (**g**–**j**) the power of the beam on the vertex positioned over the *y*-axis is decreased sequentially from 5 mW to 1 mW.

Experiments have shown that is possible to confine a RBC in its flat configuration when four beams are used for trapping [19]. We next investigate whether the same confinement of RBCs can be obtained with triple-beam OT if the foci of the three beams are positioned on the thickest portion of the RBC and arranged on the vertices of an equilateral triangle as schematically depicted in Figure 5a. 

To verify our hypothesis, we start our investigation by placing the cell in its flat configuration and displacing it along the *x*-axis between −1–1 μm, while keeping all other degrees of freedom fixed. Figure 5b shows the total optical forces acting on the center of mass of the RBC as a function of the cell’s displacement. For *x*-displacements strong force components arise along each direction. *F_z_*_,3_(*x*) derives from the scattering force generated by the lasers beams and pushes the cell in the *z*-direction. On the contrary, *F_y_*_,3_(*x*) is due to the light intensity gradient along the *y*-direction. At the origin *F_x_*_,3_(*x*) vanishes with negative gradient. This indicates that the RBC experiences a restoring force for each displacement from x_eq,3_ = 0 μm, demonstrating the transverse trapping. Fitting a line to the linear portion of *F_x_*_,3_(*x*) yields a (power normalized) spring constant of *k*_x,3_ = 0.11 pN·μm^−1^·mW^−1^ (inset Figure 5b). 

The RBC is then placed at *x*_eq,3_ in its flat configuration, and the force-displacements curve is simulated for displacements along the *y*-direction between −1 and 1 μm, Figure 5b. *F_y_*_,3_(*y*) decreases linearly within the interval examined, and vanishes with negative slope at y_eq,3_ = 0.025 μm. Again, from a linear fit to the data, we deduce the spring constant *k*_y,3_ = 0.11 pN·μm^−1^·mW^−1^ (inset of Figure 5c).

Lastly, the RBC is placed at (*x*_eq,3_, *y*_eq,3_) and the optical forces acting on the cell are calculated as a function of the displacements along the *z*-direction in the interval −1–1 μm, Figure 5d. Here, it is visible that the cell does not experiences any forces orthogonal to the beam propagation direction (*F_x_*_,3_, *F_y_*_,3_) over the entire range. *F_z_*_,3_(*z*) vanishes with negative gradient at slightly positive z (z_eq,3_ = 0.325 μm). Fitting a line to the approximately linear part of the data we obtain the (power normalized) spring constant, *k*_z,3_ = 0.09 pN·μm^−1^·mW^−1^, (inset of Figure 5d).

We then perform the analysis of the possible rotation. Initially, we placed the cell at (*x*_eq,3_, *y*_eq,3_, *z*_eq,3_) in its flat configuration and we rotate the cell between −20°–20°, where 0° is for a non-rotated cell, shown in Figure 5e. Here, is clearly visible that a stable point of equilibrium is present when the cell plane is orthogonal to the optical axis, and the characteristic torque constant is *k*_α,3_ = 0.33 pN·μm·rad^−1^·mW^−1^ (inset of Figure 5e). Therefore, we proceed to analyze the rotation around the *y*-direction in the same interval of angular displacements. As shown is Figure 5f, for any rotation from the flat configuration, the RBC experiences a restoring torque that push it back point towards 0°. From the linear fit of the linear part of graph, we obtain the (power normalized) torque *k_β_*_,3_ = 0.29 pN·μm·rad^−1^·mW^−1^ (inset Figure 5f). These results suggests that, according to our hypothesis, it is possible to stably trap a RBC with a triple-beam OT in its flat configuration. This result suggests how orientational control over the RBC can be achieved using multiple beams. The results of the numerical simulation are reported in Table 2. 

Later, we further verify the applicability of our model. To do this, we decided to test the effect of a decreasing power in of the beam, and therefore to re-orient the cell using a different power in one of the beams. Intuitively, as one of the beam powers decreases the cell should switch from a ‘flat’ to a ‘folded’ configuration to be finally trapped in a plane containing the two beams’ foci which powers are unaltered and the optical axis as the one of the beam power goes to zero. For simplicity, we decided to decrease the power of the beam located on the vertices positioned along the positive *y*-axis, Figure 5a. Doing so, the effect should manifest for rotation around the *x*-axis and for displacement along the *y*-direction only. We displace the cell along the *y*-axis keeping fixed the equilibrium coordinates and orientations obtained for a TBOT diminishing the one of the beam powers as explained previously. Figure 5g,h show there are no x- and z-component of the total force (i.e., $F_{x}\left(y\right)$ = 0 and $F_{z}\left(y\right)$ = 0) for displacements along the *y*-direction. This confirms further our expectations. As shown in Figure 5i, as the power of the beam is decreased, the gradient force generated by the beams which power is unaltered drawn the cell toward decreasing negative $y_{3,eq}$ values. Therefore, we use the new $y_{3,eq}$ to investigate the effect of the beam power on the equilibrium orientation. We therefore rotate the cell around the x-axis calculating the torque after each angular step. In Figure 5j it can be seen that $\tau_{x}\left(\alpha \right)$ show smaller and smaller slope for decreasing power, until a positive slope is found for beam power of 1 mW. Thus, if the beams powers are unbalanced to the point that the power of one of them is 1/5 of the others, the cell is repelled from the ‘flat’ configuration and trapped in the ‘folded’ configuration in a somewhat different point in space.

**Figure 6:**
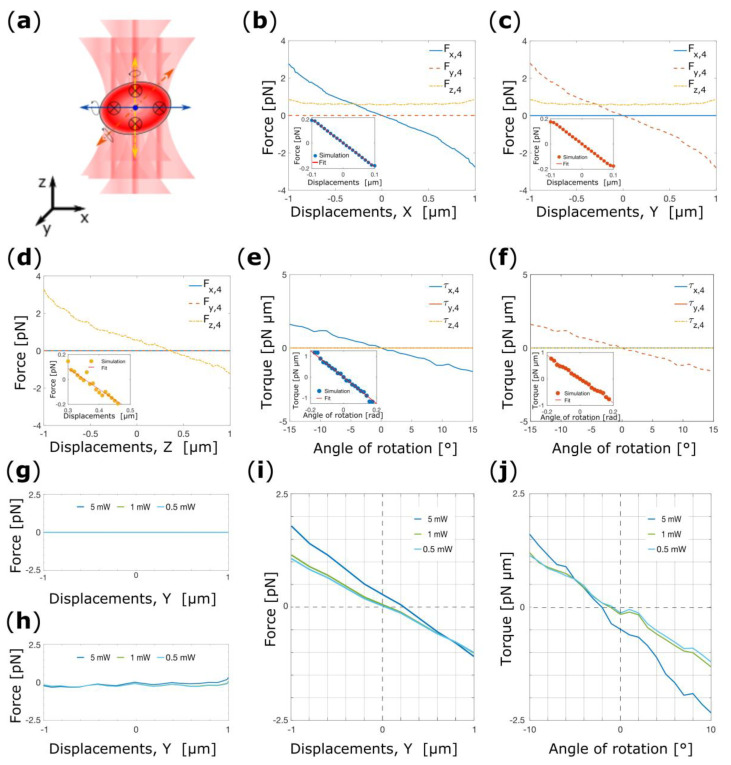
(**a**) Schematic representation of four beam OT. The arrows indicate the possible translations and rotations of the cell. Total optical force acting on the center of mass of the RBC as a function of the cell’s displacements from the center of the trap along *x* (**b**), *y* (**c**) and *z* (**d**). The optical forces are given in terms of the Cartesian components. (**e**) Torque-rotation curves for cell rotation around *x* (**e**), and *y* (**f**). In the insets are shown the linear fit to the linear portion of the force-displacements and torque-rotation curve. Force-displacements curves for cell displacements along the *x*-direction (**g**), *z*-direction (**h**), and *y*-direction (**i**) while in (**j**) is reported the torque-rotation curves. In (**g**–**j**) the power of the beam on the vertex positioned over the *y*-axis is decreased sequentially from 5 mW to 1 mW.

Now we consider a four beam OT set-up as used by Rusciano et al. for Raman Tweezers experiments [19]. In this case, the foci of the four beams are arranged on a square with vertices are positioned on the thickest portion of the cell, Figure 6a. Experiments have shown that with this set-up the RBC is confined in its flat configuration.

In accordance with the methodology used throughout, we start our investigation by analyzing the force-displacement curve for cell displacements along each Cartesian co-ordinate while keeping the cell in its flat configuration. Here, due to the symmetry of the system, the investigation of forces-displacements curve along the *y* and *x*-direction produces exactly the same outcome. For *x*-displacements, a strong force component arises along *x* and *z*, but not along *y*, Figure 6b. Analogously, if the cell is displaced along *y* optical forces are visible along *y* and *z*, but not along *x*, Figure 6c. In both cases, *F_z_*_,4_ is attributable to the scattering force which pushes the cell towards positive *z*. *F_x_*_,4_ and *F_y,_*_4_ vanish at the origin with a negative gradient, demonstrating the transverse trapping, and *x*_eq,4_ = *y*_eq,4_ = 0 μm. Fitting a line to the linear portions of the graphs we obtain the (power normalized) spring constants k_x,4_ = k_y,4_ = 0.09 pN·μm^−1^·mW^−1^ (insets Figure 6b,c). 

We then place at (*x*_eq,4_, *y*_eq,4_) and we calculate the force-displacement curve for cell displacement along the *z*-direction, Figure 6d. As expected, *F_z_*_,4_(*z*) vanishes at slightly positive z (*z*_eq,4_ = 0.350 μm) with negative gradient demonstrating the axial trapping, and the linear fit to the approximately linear range of the data produce a (power normalized) spring constant k_z,4_ = 0.08 pN·μm^−1^·mW^−1^ (inset Figure 6d). 

Lastly, we study the orientational confinement of the RBC in a four beam OT. Even in this case, the system is symmetric in respect to the *x* and *y*-direction, and the simulations effect of the beams produce exactly the same outcomes for rotation around *x* and *y*, Figure 6e,f. In both cases, a clear point of stable equilibrium arises at 0° (flat configuration). Fitting a line to the linear portion of the data we extract two symmetric (power normalized) spring constants k_α,4_ = k_β,3_ = 0.32 pN·μm·rad^−1^·mW^−1^ (insets Figure 6e,f). These results suggests that the cell is indeed confined in its flat configuration as observed in experiments [19]. The results for the FBOT are summarized in Table 3.

As last numerical experiment, we show how our model can be used by experimentalists to envisage and numerically test new experiments. As example, we choose to reproduce the experiments carried by G. Rusciano et al. where 4 beams were used to optically trap the RBC while a fifth was used to excite the Raman modes of the biomolecules of interest. Here, we are interested in predicting the available maximum power of the fifth beam before interfering with the orientational equilibrium of the cell. Given the enormous amount of available location and power for the fifth beam, for simplicity, we choose three different powers (5, 1 and 0.5 mW), and a single spatial location. We positioned the fifth beam along the positive *y*-direction at a distance of 2.76 μm from the center of mass of the cell. As for the TBOT, this simplifies the successive analysis. Again, we begin our analysis by displacing the cell along the *y*-direction starting from the equilibrium coordinates found for a FBOT. No force components are present along the a- and *z*-directions as shown in Figure 6g,h. On the contrary, as the power of the beam is increased a net force is generated along the *y*-direction that drawn the cell toward a positive $y_{4,eq}$, while the $y_{4,eq}$ is left unchanged if the power of the fifth beam is 1 or 0.5 mW Figure 5i. Successively, for analyzing the rotational stability we use the new $y_{4,eq}$. In Figure 5j it can be seen that the rotational equilibrium of the cell is perturbed only when the power of the fifth beam is 5 mW. These results are in complete agreement with what has been experimentally shown by Rusciano et al. In fact, researchers stably trap and healthy RBC using four beams with 10 mW of power while a fifth beam with 1 mW of power were used to excite the Raman modes of the macromolecules of interest. 

## 4. Conclusions

This work presents a numerical investigation of the optical forces and torques acting on a healthy RBC in its biconcave disk conformation when trapped by a multiple beam OT in the ray optics regime. Care was taken to meticulously analyze how the complex biconcave disk shape influence the ray paths, the optical forces and torques, and ultimately the equilibrium configuration of a RBC within an optical tweezers. Moreover, our numerical investigation shows that a different number of beams can be used to balance the optical torques experienced by a RBC, and ultimately to confine the cell in specific orientation depending on the specific requirements of the researchers. One or two beams can be used to orient the RBC parallel to the optical axis, while increasing the number of beams to three or four the cell can be constrained to be perpendicularly oriented in respect to the optical axis. Nonetheless, the geometry and powers with which the beams are arranged are of particular importance in determining the orientational stability. Even if we show that a stable RBC confinement can be obtained with up to four trapping beams, we cannot exclude the possibility to confine the RBC with five or more beams with specific orientation and position if properly arranged. In conclusion, our analysis can be straightforwardly adopted by experimentalist to investigate and estimate both quantitative parameters for optical trapping, such as the trap stiffness, and qualitative behavior such as cell orientation before performing experiments.

## Figures and Tables

**Table 1:** Values of trap stiffnesses, rotational equilibrium and rotational trap stiffness for an RBC trapped by a two-beams optical tweezers. The stiffnesses are normalized over the total power.

Equilibrium Position (μm)	Stiffness (pN·μm^−1^·mW^−1^)	Rotational Equilibrium (°)	Rotational Stiffness (pN·μm·rad^−1^·mW^−1^)
*x*_eq,2_ = 0	k_x,2_ = 0.15	90	k_α,2_ = 0.37
*y*_eq,2_ = 0	k_y,2_ = 0.24	0	0
*z*_eq,2_ = −0.230	k_z,2_ = 0.05	0	k_γ,2_ = 1.73

**Table 2:** Values of the spatial equilibrium, spring constants, rotational equilibrium and torque constants for an RBC trapped by a three-beams optical tweezers.

Equilibrium Position (μm)	Stiffness (pN·μm^−1^·mW^−1^)	Rotational Equilibrium (°)	Rotational Stiffness (pN·μm·rad^−1^·mW^−1^)
*x*_eq,3_ = 0	k_x,3_ = 0.11	0	k_α,3_ = 0.33
*y*_eq,3_ = 0.025	k_y,3_ = 0.11	0	k_β,3_ = 0.29
*z*_eq,3_ = 0.325	k_z,3_ = 0.09	0	0

**Table 3:** Values of trap stiffnesses, rotational equilibrium and rotational trap stiffness for an RBC trapped by a four-beams optical tweezers. The stiffnesses are normalized over the total power.

Equilibrium Position (μm)	Stiffness (pN·μm^−1^·mW^−1^)	Rotational Equilibrium (°)	Rotational Stiffness (pN·μm·rad^−1^·mW^−1^)
*x*_eq,4_ = 0	k_x,4_ = 0.09	0	k_α,4_ = 0.32
*y*_eq,4_ = 0	k_y,4_ = 0.09	0	k_β,3_ = 0.32
*z*_eq,4_ = 0.350	k_z,4_ = 0.08	0	0

## Data Availability

Data and codes are available from authors upon reasonable request.
